# Novel Positive-Sense, Single-Stranded RNA (+ssRNA) Virus with Di-Cistronic Genome from Intestinal Content of Freshwater Carp (*Cyprinus carpio*)

**DOI:** 10.1371/journal.pone.0029145

**Published:** 2011-12-16

**Authors:** Ákos Boros, Péter Pankovics, Peter Simmonds, Gábor Reuter

**Affiliations:** 1 Regional Laboratory of Virology, National Reference Laboratory of Gastroenteric Viruses, ÁNTSZ Regional Institute of State Public Health Service, Pécs, Hungary; 2 University of Edinburgh, Edinburgh, Scotland, United Kingdom; Chungbuk National University, Republic of Korea

## Abstract

A novel positive-sense, single-stranded RNA (+ssRNA) virus (Halastavi árva RNA virus, HalV; JN000306) with di-cistronic genome organization was serendipitously identified in intestinal contents of freshwater carps (*Cyprinus carpio*) fished by line-fishing from fishpond “Lőrinte halastó” located in Veszprém County, Hungary. The complete nucleotide (nt) sequence of the genomic RNA is 9565 nt in length and contains two long - non-in-frame - open reading frames (ORFs), which are separated by an intergenic region. The ORF1 (replicase) is preceded by an untranslated sequence of 827 nt, while an untranslated region of 139 nt follows the ORF2 (capsid proteins). The deduced amino acid (aa) sequences of the ORFs showed only low (less than 32%) and partial similarity to the non-structural (2C-like helicase, 3C-like cystein protease and 3D-like RNA dependent RNA polymerase) and structural proteins (VP2/VP4/VP3) of virus families in *Picornavirales* especially to members of the viruses with dicistronic genome. Halastavi árva RNA virus is present in intestinal contents of omnivorous freshwater carps but the origin and the host species of this virus remains unknown. The unique viral sequence and the actual position indicate that Halastavi árva RNA virus seems to be the first member of a new di-cistronic ssRNA virus. Further studies are required to investigate the specific host species (and spectrum), ecology and role of Halastavi árva RNA virus in the nature.

## Introduction

Picorna-like viruses are a loosely defined group of non-enveloped, positive-sense single-stranded RNA (+ssRNA) viruses that are major pathogens of humans, animals, insects and plants [1]. These viruses have similar genome features and several conserved protein domains. In 2009, a new order was detached within picorna-like viruses. The order *Picornavirales* contains several important viruses grouped into 5 families: *Dicistroviridae*, *Iflaviridae*, *Marnaviridae*, *Picornaviridae* and *Secoviridae* [1], [2].

Based upon the genome organization non-enveloped, positive-sense single-stranded RNA (+ssRNA) viruses have two groups: viruses with mono- or di-cistronic genome characteristics. Iflaviruses, marnaviruses, picornaviruses and secoviruses have monocistronic genome structure with a large open reading frame (ORF) coding a single polyprotein. However, dicistroviruses - the name (dicistro) is derived from the characteristic di-cistronic arrangements of the genome - have two non-overlapping large open reading frames (ORF1 and ORF2). In dicistroviruses, the structural proteins are located at the 3′ end (ORF2) rather than the 5′ end as found in iflaviruses, marnaviruses, picornaviruses and secoviruses. The most outstanding characteristics of dicistroviruses is that they have two internal ribosome entry site (IRES) elements, one for translation of the ORF1 replicase (including 2C-like helicase (Hel), VPg, 3C-like protease (Pro) and 3D-like RNA dependent RNA-polymerase (Pol) in order Hel-(VPg)_x_-Pro-Pol module of conserved replication domains) and the other – the IGR (intergenic region) - IRES - for translation of the capsid proteins (VP2-VP4-VP3 and VP1) [3]. Recently, it is proposed that dicistrovirus Plautia stali intestine virus (PSIV) ORF1 polyprotein precursor contains all proteins (2A–C, 3A–D) and in a same order as in picornaviruses [4].

Members of dicistroviruses have originally been identified in aphids, leafhoppers, flies, bees, ants, silkworms and shrimps [5]. Some of them are pathogenic to arthropods such us honey bees and shrimp and to insect pests of medical and agricultural importance [5]. Recently, several viruses with di-cistronic genome organization were identified from seawater virus communities. Di-cistronic RNA viruses (marine RNA virus JP-A and JP-B) have been detected in marine environmental (seawater) samples with unknown host origin [6], [7] and in marine protists including diatoms [8], [9], [10] and fungoid protist [11]. These viruses are not members of any currently defined virus families and the taxonomic positions of these marine di-cistronic viruses are not yet determined; however, there are two proposed family names – “Bacillariornaviridae” and “Labyrnaviridae” – for diatom viruses (*Rhizosolenia setigera* RNA virus (RsetRNAV), *Chaetoceros socialis f. radians* RNA virus (CsfrRNAV) and *Chaetoceros tenuissimus* RNA virus (CtenRNAV) and fungoid protist virus (*Aurantiochytrium* single-straded RNA virus (AsRNAV): previously designated as *Schizochytrium* single-stranded RNA virus (SssRNAV), respectively [10], [12]. Interestingly, mono-cistronic RNA virus – *Heterosigma akashiwo* RNA virus (HaRNAV), a prototype member of *Marnaviridae* – is infecting unicellular bloom-forming raphidophyte alga [13], [14]. Recently, dicistro-like viruses have been identified in human intestinal tract by metagenomic survey [15].

The first report of metagenomic analyis of RNA viruses in fresh water lake was published in 2009 [16]. This study reports a high number and high diversity of RNA viruses in a freshwater pond. Here we report the serendipitous identification and characterization of a novel possibly non-enveloped, positive-sense single-stranded RNA (+ssRNA) virus with di-cistronic genome structure originating from unknown host(s) from intestinal content of freshwater carp (*Cyprinus carpio*) in Hungary.

## Results

### Identification and general features of a novel viral RNA genome sequence in intestinal content of carp

**Figure 1:**
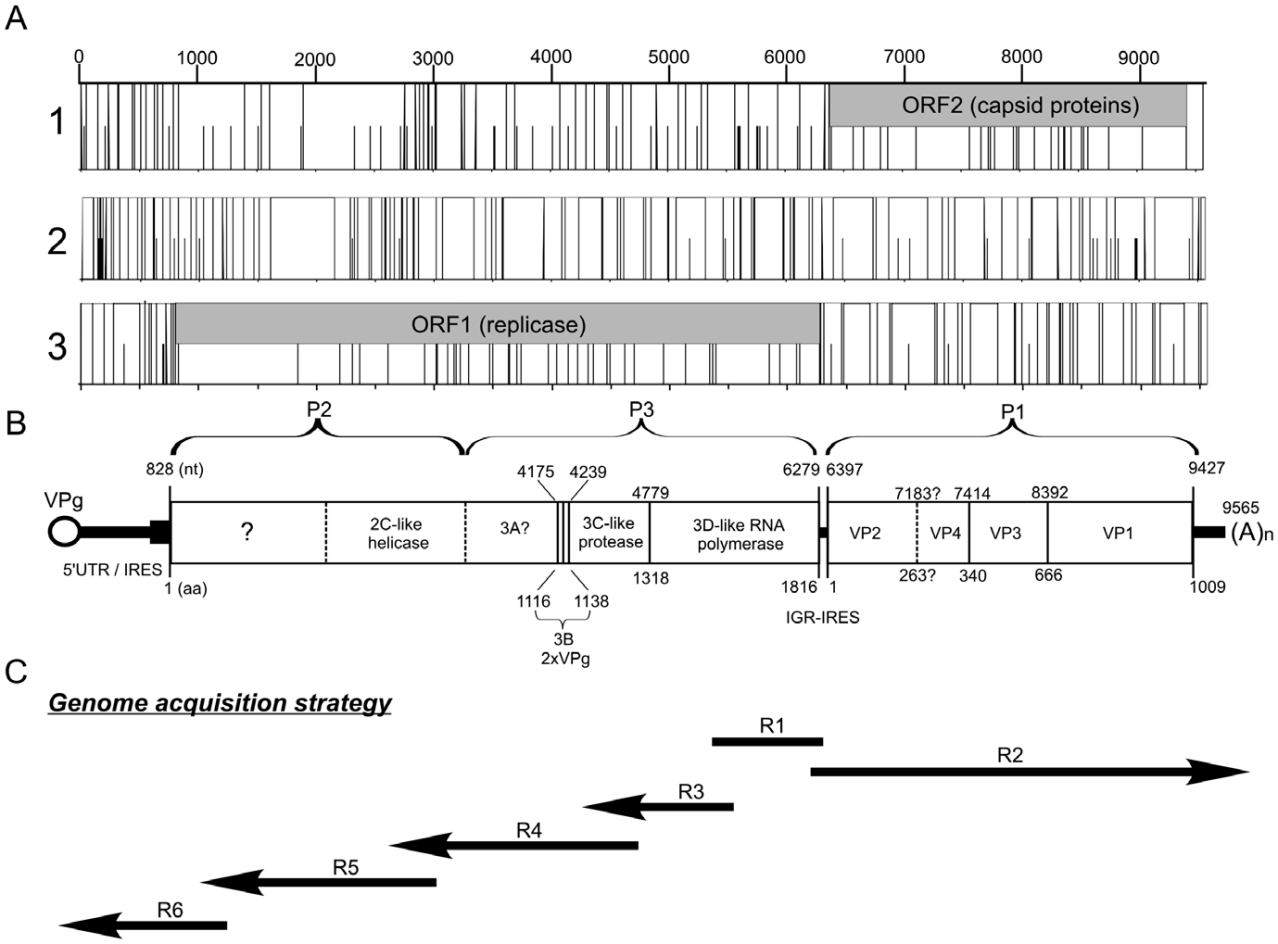
Analysis of Halastavi árva RNA virus (HalV) complete genome (9565 nt) for putative open reading frames. In the ORF map (Fig. 1A) for each reading frame (1, 2 and 3), potential start codons (AUG) are shown with a half-height line and stop codons (UGA, UAA, UAG) are shown by full-height lines. Predicted and recognizable viral proteins and other genomic features (UTR = untranslated region; IGR = intergenic region) are noted (Fig. 1B). Numbers for nucleotide (nt; upper number) and amino acid (aa; lower number) sequences indicate the first sequence positions of the next regions. Cleavage sites were not experimentally determined but estimated by alignment of deduced amino acid sequences (see text for more detail). Fig. 1C shows the genome acquisition strategy using 3′/5′-RACE method.

Using non-human entero-R/F screening primers (Table 1) an approximately 1000-nt-long single PCR-product was seen from a carp intestinal content in agarose gel (Fig. 1C). Homologue nucleotide sequence was not found in GenBank. However, significant amino acid sequence identity and characteristic amino acid sequence motifs (YGDD and FLKR) of RNA dependent RNA-polymerase gene (replicase) of several members of picorna-like superfamily were found including dicistroviruses, picornaviruses and secoviruses with top matches to marine JP-B virus (EF198242; *E* value = 4×10^−14^, identities = 64/179; 36%) using BLASTp searches of the NCBI database. The novel genome sequence was provisionally named as Halastavi árva (in Hungarian = “fishpond orphan” in English) RNA virus (abbreviation: HalV).

**Table 1:** List of primers used in this study for detection and characterization.

Reaction type	Reaction No.	Primer name	Primer location (nt)	Primer sequence
RT-PCR	R1	Non-Human Entero-5′UTR-R	6468-6451?	5′-CRG AGC TAC CAC TGG GGT-3′
		Non-Human Entero-5′UTR-F	5458-5475	5′-GGG AGT AGT CCG ACT CCG-3′
3′ RACE	R2	s1	6071-6091	5′-TAG AGA GAT GGC CAA TTA CAC-3′
		s2	6575-6594	5′-TAC CAT CAG AGA TGG AAG CG-3′
5′ RACE	R3	as1a	5685-5665	5′-GTG TTG GGA ATT TGA TGC CTT-3′
		as1b	5593-5573	5′-GGT CGA AGT GCT GCG TCT ATA-3′
		as1c	5570-5551	5′-ACT GGG GCT TTG CGA CGA AT-3′
	R4	as2a	5066-5047	5′-CTC CTC AAT TGT GAG AAC GC-3′
		as2b	4908-4888	5′-GTT TTA GGA TGG CTG GGG CTG-3′
		as2c	4587-4570	5′-CAC CAA ACG TGG TTT CGG-3′
	R5	as3a	4044-4028	5′-TGC GCA TGG CTG GAG TT-3′
		as3b	3436-3418	5′-TCA GGT GTG ATC TCG AGA G-3′
		as3c	3293-3275	5′-GAT GAT AGA TGG AAT GTC C-3′
	R6	as4a	2663-2646	5′-AGA GAG TGT AGC CTT GGG-3′
		as4b	2237-2220	5′-GTC CGC TGT GAC GTA CAC-3′
		as4c	1664-1646	5′-GCC GAA TGA TTC GAA CAG T-3′
3′/5′ RACE	all	Oligo dT anchor		5′-GAC CAC GCG TAT CGA TGT CGA C T_(16X)_ V-3′
		PCR anchor		5′-GAC CAC GCG TAT CGA TGT CGA C-3′

Fig. 1C shows the positions of Reaction No. R1-R6 in genome acquisition strategy using 3′/5′-RACE method.

No significant sequence match was found in GenBank based upon the complete nucleotide sequence. The Halastavi árva RNA virus genome is 9565 nt in length (Fig. 1), excluding the polyadenylate - poly(A) - tail, with 827 nt 5′untranslated region (UTR) followed by 2 predicted non-overlapping ORFs of 5451 nt (ORF1, nt position 828 to 6278) and 3030 nt (ORF2, nt position 6397 to 9426) separated by an intergenic region (IGR) of 118 nt (Fig. 1A). ORF2 is followed by a 3′UTR of 139 nt (nt position 9427 to 9565). Two large ORFs were found which encode potential polyprotein precursors of 1816 aa (ORF1) and 1009 aa (ORF2). No large ORFs were found in the inverse orientation suggesting that Halastavi árva RNA virus is a positive-strand RNA virus with dicistronic genome organisation.

### Analysis of non-structural region (ORF1)

ORF1 possesses an AUG codon at nt 828 and extends to nt 6278 (Fig. 1). ORF1 encodes an 1816 aa protein. Comparison to know viral sequences using the conserved domain search of the NCBI database shows that the complete protein sequence predicted to be encoded by ORF1 of Halastavi árva RNA virus contains several conserved sequence motifs (Table 2) and the Hel-(VPg)_x_-Pro-Pol module of conserved replication domains. Except the approximately 350 aa 5′-end, which has no sequence match in GenBank, the ORF1 has characteristic aa sequences of 2C-like helicase/NTP-binding protein (motifs: GKS, YDDF, KATLSEK, starting at aa residues 522, 575 and 608 in ORF1), 3C-like cystein protease (GDCG, ILGIHGA; starting at aa residues 1253 and 1270) and 3D-like RNA-dependent RNA polymerase (KDERR, VGINPDSAEW, LGDY, PSG, YGDD, FLKR and APL; starting at aa residues 1488, 1543, 1590, 1634, 1676, 1727 and 1741). The “picorna-like” 2A motif NPGP was not found. Potential tandem repeat sequence Q/HCAIGLFVKDQ/SCLMNKHSLDQ/L was detected at aa position 1116 and 1127 (between 2C-like cystein protease and 3C-like RNA polymerase) corresponding to potential two short (11 aa each) VPg (3B) sequences (Fig. 1B). These small virus proteins could covalently link to the 5′ end of the genome. The amino acid sequences that are most similar to complete ORF1 of Halastavi árva RNA virus were the non-structural protein regions of ant virus Solenopsis invicta virus 1 (YP_164440; *E* value = 5×10^−58^, identities = 238/743; 32%) and the shrimp virus Taura syndrome virus (AAT81157.2; *E* value = 1×10^−56^, identities = 264/872; 30%). Detailed amino acid similarity analysis of ORF1 regions are shown in Table 2. The predicted 3C mediated cleavage sites are Q/T (at aa positions 1317/1318) between 3C and 3D (Fig. 1B).

**Table 2:** Percental amino acid identity of the Halastavi árva RNA virus protein regions compared to the GenBank sequences using blastp (see details in Materials).

		Halastavi árva RNA virus					
		ORF1 (replicase)			ORF2 (capsid proteins)		
		2C-like helicase	3C-like protease	3D-like polymerase	VP2/VP4	VP4/VP3	VP3/VP1
		1–702 (702 aa)	703–1317 (615 aa)	1318–1816 (498 aa)	1–340 (340 aa)	264–666 (403 aa)	341–1009 (668 aa)
Sequences producing significant blastp alignments	Putative conserved domains (*E* value)	P-loop NTPase superfamily (2×10^−18^)	Peptidase C3/C4 superfamily (0.01/0.02)	RT-like superfamily (4×10^−44^)	Spherical virus-type peptidase (0.13)	Dicistro-VP4 (5×10^−3^)	Picornavirus capsid protein domain-like/rhv-like superfamily (2×10^−19^)
**Taura syndrome virus** [AAY89096] (Host: *Penaeus vannamei*/whiteleg shrimp)	Identity (*E* value)	**30% (96/319, 3×10^−24^)**					
Himetobi P virus [BAD27584] (Host: *Nilaparvata lugens*/brown planthopper)	Identity (*E* value)	25% (97/390, 1×10^−21^)					
**Aphid lethal paralysis virus** [AAN61470] (Host: *Rhopalosiphum padi*/small-grain aphid)	Identity (*E* value)		**28% (50/179, 2×10^−6^)**				
Triatoma virus [AAF00472] (Host: *Triatoma infestans*/vinchuca)	Identity (*E* value)		24% (68/286, 3×10^−4^)				
**Solenopsis invicta virus 1** [AAU85375] (Host : *Solenopsis invicta*/red imported fire ant)	Identity (*E* value)			**33% (161/495, 1×10^−49^)**			
Acute bee paralysis virus [AAG13118] (Host: *Apis mellifera*/black bee)	Identity (*E* value)			30% (143/471, 2×10^−48^)			
**Himetobi P virus** [BAD27585] Host: *Nilaparvata lugens*/brown planthopper	Identity (*E* value)				**30% (63/211, 3×10^−12^)**		
Aphid lethal paralysis virus [AAN61471] (Host: *Rhopalosiphum padi*/small-grain aphid)	Identity (*E* value)				25% (79/312, 4×10^−10^)		
**Marine RNA virus JP-A** [ABQ50600] (Host: Unknown)	Identity (*E* value)					**28% (94/331, 1×10^−22^)**	
Marine RNA virus JP-B [ABQ50602] (Host: Unknown)	Identity (*E* value)					25% (95/375, 7×10^−17^)	
**Cricket paralysis virus** [AAF80999] (Host: *Teleogryllus commodus*/australian field cricket)	Identity (*E* value)						**25% (93/367, 2×10^−16^)**
Pteromalus puparum small RNA-containing virus [ACG60668] (Host: *Pteromalus puparum*/endoparasitoid wasp)	Identity (*E* value)						24% (119/503, 4×10^−10^)

Putative conservative domains represent the identified conservative domains using NCBI cddsearch in the Pfam and CDD databases. In each search two viral sequences were chosen: one with the lowest *E* value (in bold) and one with the longest alignment (in simple letters). In the first column (“Sequences producing significant blastp alignments”) names, protein accession numbers, and the host species of the closest viral sequence match were shown. The rows of “Identity (E-value)” represent the percental amino acid identity (underlined) compared to the sequences in the first column, and in brackets the identical amino acids/alignmented sequence length takes into consideration by the blastp and the related E-value.

### Analysis of structural region (ORF2)

ORF2 is in a different frame to ORF1 (Fig. 1A). It possesses an AUG codon at nt 6397 and extends to nt 9426. ORF2 encodes a 1009 aa protein (Fig. 1B). This ORF2 protein length is between the shortest (765 aa, Kashmir bee virus, AAR19088) and the longest (1138 aa, *Aurantiochytrium* single-stranded RNA virus, YP_398835) dicistrovirus ORF2. Recognizable putative picornavirus-like capsid protein domains were identified by the conserved domain search of the NCBI database (Table 2). Multiple alignments are show conserved amino acids (underlined: GRLI, LRIPF, FGFSSP, DEM and YWAGSI, VATPFHAGRLVLAYVP, VWD, GE, DDFSF) on the Halastavi árva RNA virus with the capsid proteins (VP2, VP4 and VP3) of viruses in family *Dicistro*- and *Picornaviridae*. In addition, an assumed spherical virus-type peptidase (Conserved domains database, pfam12264) with high *E* value (0.13) was also identified at the N-terminal end (from 75–250 aa in VP2) of the putative ORF2 capsid-polyprotein (Table 2). There was no sequence hits on the predicted VP1 region at the 3′ end of the ORF2. The sequences that are have recognizable similarity to complete ORF2 VP2-VP4-VP3 of Halastavi árva RNA virus were the structural protein regions of Cricket paralysis virus (NP_647482; *E* value = 8×10^−32^, identities = 166/664; 25%) and Drosophila C virus (NP_044946; *E* value = 8×10^−31^, identities = 161/688; 24%). The amino acid sequence identity of Halastavi árva RNA virus ORF2 to marine RNA virus JP-A is 28% (*E* value = 2×10^−22^, identities = 94/331). Detailed amino acid similarity analysis of ORF2 regions (VP2-VP4; VP4-VP3; VP3-VP1) are shown in Table 2.

Cleavage sites of the ORF2 encoded polypeptide were not experimentally determined but estimated by alignment of deduced amino acid sequences. The putative protease-mediated cleavage sites of ORF2 polyprotein are RAFGF/SSPPD (highly conserved motif among dicistroviruses at aa position 339/340) and ESMQ/DPY (at aa position 665/666) between capsid protein VP4/VP3 and VP3/VP1 in Halastavi árva RNA virus, respectively. The predicted amino acid length of VP3 and VP1 are 326 aa and 344 aa. VP2+VP4 are 339 aa long. The cleavage site between VP2 and VP4 is probably between aa260 or aa280, maybe at position aa262/aa263 (Q/T). Based upon this calculation the predicted VP2 and VP4 minor capsid proteins are 262 and 77 amino acid-long, respectively.

### Analysis of the non-coding region: untranslated regions (UTRs) and intergenic region (IGR)

ORFs 1 and 2 represent 88.7% of the RNA genome. The other 11.3% consists of non-coding regions (UTRs and IGR). The 5′UTR is 827 nts in length and it predicted an extensive secondary structure with several long base-paired hairpins (data not shown). Between ORF1 replicase and ORF2 capsid coding regions Halastavi árva RNA virus has a predicted IGR of 118 nts. The exact length of the functional IGR and the secondary structure of the IGR-IRES remained unknown. Conserved dicistrovirus IGR-IRES-like structural elements in which they were previously reviewed by Nakashima & Uchiumi (2009) were not detected. The length of the 3′UTR in dicistroviruses varies from about 150 to 500 nucleotides. Halastavi árva RNA virus 3′UTR is short (139 nt excluding the poly(A) tail) and has an extensive predicted secondary structure consisted of “T”-like double-stranded hairpins (figure not shown). Similar non-coding sequences had not been found in GenBank.

### Phylogenetic analysis

**Figure 2:**
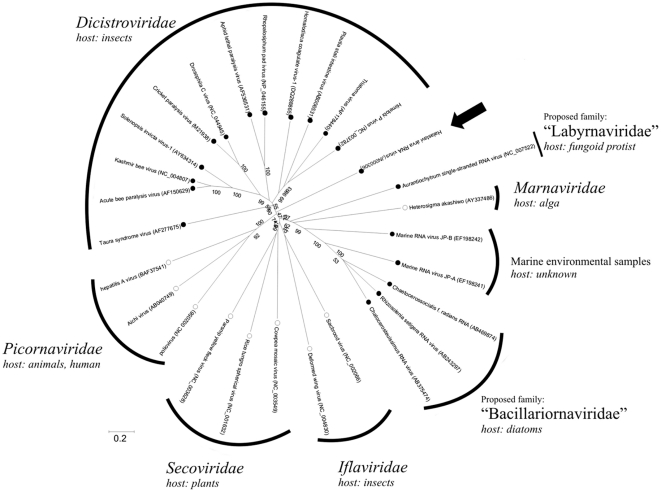
Phylogenetic tree of nonstructural region. Phylogenetic analysis based upon the amino acid sequences of complete nonstructural region (including 2C-like helicase, 3C-like protease and 3D-like RNA dependent RNA-polymerase) between the members of the order *Picornavirales* (*Dicistroviridae*, *Iflaviridae*, *Marnaviridae*, *Picornaviridae* and *Secoviridae*), marine positive-sense single-stranded RNA (+ssRNA) viruses with di-cistronic genome organization and Halastavi árva RNA virus (JN000306) identified in intestinal content of freshwater carp (*Cyprinus carpio*) in Hungary. Black (•) and white (○) circles represent viruses with di- and mono-cistronic genome structure. Specific hosts – if known - are shown in relation with the virus family name. Halastavi árva RNA virus is indicated by black arrow.

**Figure 3:**
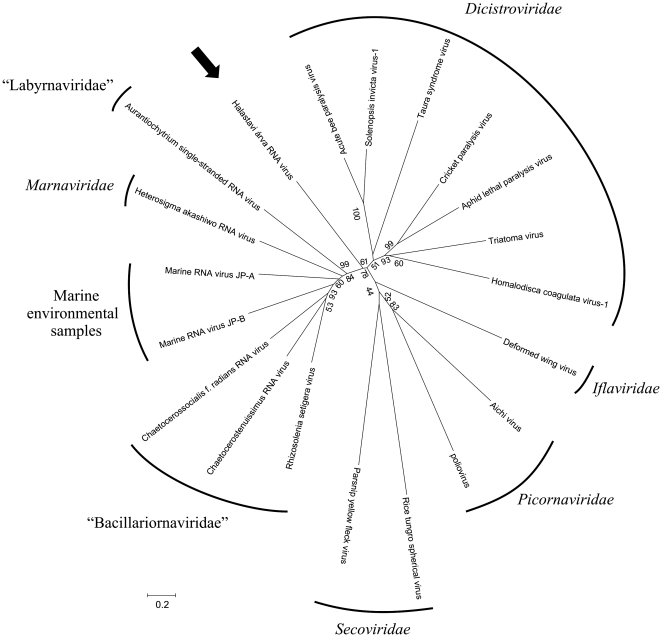
Phylogenetic tree of structural region. Phylogenetic analysis of amino acid sequences of complete capsid (VP2/VP4/VP3/VP1) region of representative members in the order *Picornavirales*. The accession numbers of the reference strains are showed in Figure 2. Halastavi árva RNA virus (JN000306) is indicated by black arrow.

Figure 2 and 3 shows the phylogenetic analysis of the amino acid sequence of the complete ORF1 (Fig. 2) and complete ORF2 (Fig. 3) regions of Halastavi árva RNA virus and representative members of the highly diverse *Picornavirales* order. Halastavi árva RNA virus showed no close relationship to any existing viruses, an observation consistent with the lack of close resemblance of its genome architecture to that of other known RNA viruses.

### Nucleotide and dinucleotide composition

**Figure 4:**
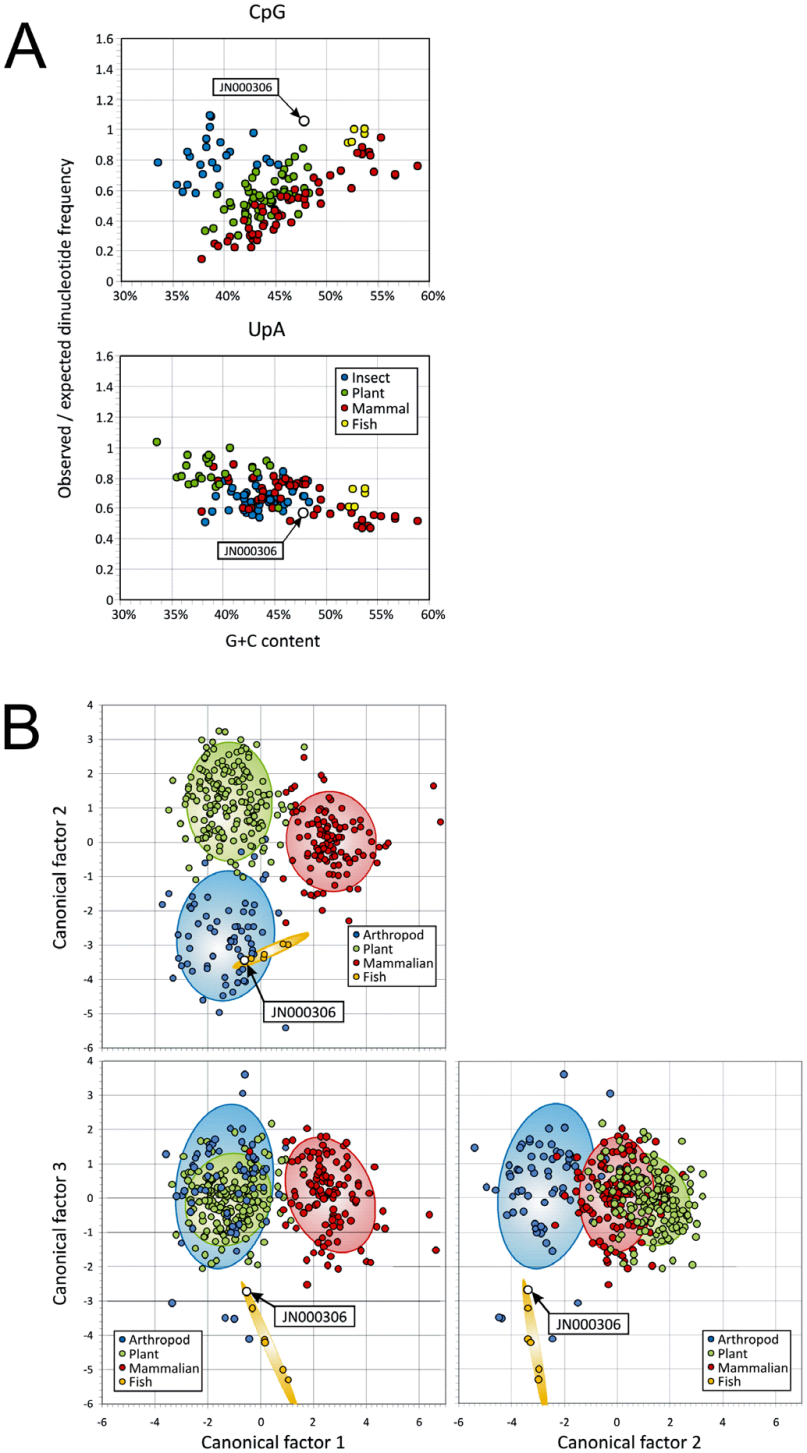
Nucleotide composition analysis of Halastavi árva RNA virus. (A) Frequencies of CpG and UpA dinucleotides in picorna-like viruses from different hosts expressed as observed-to-expected ratios (y-axis). (B) Projections of the first three (most significant) canonical factors that differentiate host origins of the control sequences using mononucleotide dinucleotide frequencies. Points represent values for individual sequences, with 95% confidence ellipses positioned around the centroid of each group [17]. Halastavi árva RNA virus (JN000306) is labeled by black arrows.

The base composition of Halastavi árva RNA virus is 26.6% A, 27.4% C, 20.3% G, and 25.7%U; this results in a G+C of 47.7%. In common with other RNA viruses (and host genomes), Halastavi árva RNA virus shows a variety of under- and over-representations of dinucleotide frequencies compared to those expected from its mononucleotide base composition (Fig. 4A). For example, frequencies of the UpA dinucleotide were only 57% of the expected value, clustering closely with other picorna-like RNA viruses. However, in contrast to most picorna-like viruses infecting mammals and plants, Halastavi árva RNA virus showed no under-representation of the CpG dinucleotide with an observed/expected ratio of greater than 1. In this respect, its genome composition was more similar to those of insect and fish viruses (blue and yellow data points).

Nucleotide composition analysis (NCA) was used to predict the possible host for Halastavi árva RNA virus. In contrast to previous use of this method [17], an additional host group (fish) was included as a further control set in the analysis. Host predictions of the control sequences by showed 95% concordance with assignments based on sequence annotations, including the reliable differentiation of fish-derived sequences from insect viruses (that also lack the CpG deficiency observed in animal and plant viruses; Table S1). Using this method, the host assignment of Halastavi árva RNA virus was fish. Figure 4B shows a series of projections of the first three canonical factors (CFs) calculated by discriminant analysis. While differentiation of fish viruses from insect-derived viruses was limited using CF1 (which was primarily based of CpG; data not shown), CF3 showed strong differentiation of these two hosts, creating two distinct clusters (blue and yellow ellipses); Halastavi árva RNA virus fell into the fish-derived cluster consistent with its assignment by discriminant analysis.

### Detection of Halastavi árva RNA virus in further carp sample

Using specific primers designed for Halastavi árva RNA virus 2C-like helicase/3A? region (between nt positions 3265 and 3714) a specific 450 nt long PCR-product was detected for Halastavi árva RNA virus by RT-PCR and sequencing in the intestinal content of a second carp, too, fished from the same fishpond at the same time. The two nucleotide sequences were identical.

## Discussion

A novel – possibly non-enveloped - positive-sense single-stranded RNA (+ssRNA) virus (Halastavi árva RNA virus; HalV) with di-cistronic genome organization was identified and characterized originated from the intestinal content of freshwater carp (*Cyprinus carpio*) in Hungary. Based upon the results of the sequence- and phylogenetic analysis this virus belongs to the order *Picornavirales*. Halastavi árva RNA virus has no nucleotide sequence match in GenBank based upon the complete nucleotide sequence. Halastavi árva RNA virus has only low and partial amino acid sequence identity to known virus families in *Picornavirales*. In addition, the putative nonstuctural protein domains and capsid proteins show a mosaic pattern of relationships with sequences from viruses in this order especially in family *Dicistroviridae*. However, Halastavi árva RNA virus is different from the presently known virus families according to the possible host organism; the nucleotide/amino acid sequence identity; the possible unique structures of UTR/IGR and the AU/GC ratios compared to other viruses in *Picornavirales*. These data suggests that Halastavi árva RNA virus seems to be the first member of a possible new – previously unknown – family of ssRNA viruses.

Inferring the host of genetically distinct viruses is problematic especially if they are found in feces. Feces are known to contain viruses that infect host cells and/or bacteriophages, as well as viruses of dietary origins from consumed plants, insects, and animals [17]. As freshwater carp (*Cyprinus carpio*) are omnivorous, host assignment of Halastavi árva RNA virus recovered from intestinal contents of these fishes is particularly problematic. As a specific example, we have previously shown that viruses recovered from faeces of a child from Afghanistan which showed an unexpected genome organizations similar to insect dicistroviruses, could be predicted to have an insect host by NCA, in this case also potentially deriving from a dietary source [17]. NCA was used in the current study in order to predict host origins for Halastavi árva RNA virus by including a further group, fish (primarily containing available sequences from nodaviruses) into the analysis. The resolution of this method was clearly limited by the low number of available viral sequences from fish sufficiently long for robust composition analysis and the restricted diversity of the sequence used. Consequently, the conclusion for a fish host origin (in contrast to insect or potentially other arthropod or nematode origins) has to be regarded as provisional pending the availability of further fish-derived sequences that can be used by NCA as controls. The future incorporation of several picornaviruses recently identified in fish (Knowles et al., personal communication) will be of value in this regard. Therefore, there was no evidence of a close relationship with viruses infecting humans or other mammals among the order *Picornavirales*. Further specific studies are required to investigate the specific host species (and spectrum), ecology and role of Halastavi árva RNA virus in the nature.

The AU ratios of the three diatom viruses with ssRNA genome range 60.4% to 63.7%. In insect dicistroviruses AU ratios range 60 to 64%, while that of HaRNAV and SssRNAV are much lower; 53.1% and 50.2%, respectively. Interestingly, the base composition of Halastavi árva RNA virus is more similar (52.3%) to this last group. On the other hand, Halastavi árva RNA virus genome structure appears to have a polycistronic genome organization similar to that found in viruses in family *Dicistroviridae*. Several of these viruses contain internal ribosome entry site (IRES) that position the ribosome on the genome, actuating translation initiation even in the absence of known canonical initiation factors [3]. The exact secondary structures (and functional parts) of these untranslated regions (5′UTR, 3′UTR and a rather short IGR) of Halastavi árva RNA virus remained unknown. Insect origin conserved dicistrovirus IGR-IRES-like structural elements, previously reviewed by Nakashima and Uchiumi [3], were not detected. This observation implies that there is probably other IRES-like RNA structure in Halastavi árva RNA virus.

During the conserved domain search, a putative spherical virus-type peptidase was identified at the N-terminal end of the ORF2 polypeptide. This type of peptidase is responsible for the cleavage of the viral polyprotein into individual proteins in Rice tungro spherical virus [18], although this enzyme coded in the non-structural region. This finding brings up the possibility of the putative dual function of the VP2 (structural protein with enzymatic function). Based upon the current knowledge, there is no information regarding to the presence of a peptidase in the capsid proteins among picornaviruses. However, the peptidase function could be important during the cleavage of the capsid-polyprotein. The presence of the 5′UTR-IRES and IGR-IRES in the viruses with di-cistronic genome organization could suggest the distinct initiation time of the ORF1 and ORF2 transcriptional processes. In this case the peptidase function of the N-terminal end of the capsid-polyprotein is useful for the capsid protein cleavage. To support this hypothesis we found the presence of the same putative spherical virus-type peptidase in the N-terminal end of the Himetobi P dicistrovirus capsid polypeptide (BAD27585.1, 88–248 aa) by conservative domain search (*E* value = 10^−5^). The verification of the presence of this putative peptidase at the N-terminal capsid polyprotein in viruses with di-cistronic genome organization warrants future investigations.

Unexpectedly high number of known and novel viruses particularly +ssRNA viruses were identified in marine and recently in fresh water samples by metagenomic methods [16]. In addition, there are likely to be many, yet undiscovered viruses with di-cistronic genome structure. This is the first report of identification and complete genetic characterization of a virus with di-cistronic genome organization from freshwater communities, possibly a fish, which if confirmed will represent the first report of this family of virus infecting a vertebrate species.

## Materials and Methods

### Sample collection

Intestinal contents were freshly collected from the intestine of two freshwater carps (*Cyprinus carpio*) during fish processing. No further samples are available from fishes. Carps were fished by line-fishing from fishpond “Lőrinte halastó” located in Veszprém County, Hungary, in Apr 18, 2010. The specimen was stored at −20°C until RNA isolation.

### RNA isolation, RT-PCR

RNA was extracted from 150 µl intestinal content suspension (35–40 v/v% in 0,1 M phosphate buffered saline) using TRIzol reagent according to the manufacturer's instructions (Invitrogen, Carlsbad, CA). Generic Non-HumanEntero-5′UTR PCR primers (Table 1) were used for the detection of any picornaviruses by RT-PCR method. The generic primers were designed for a conservative nucleotide sequence of the 5′UTR of the known non-human, non-simian enterovirus reference strains obtained from GenBank. All reagents were purchased from Promega (Madison, WI) unless otherwise specified. The cDNA synthesis was carried out in 50 µl final volume containing 5 µl of RNA extract; 10 mM dNTP, 5 µl 10× PCR buffer (Sigma, St Louis, MI), 1 µl 25 mM MgCl_2_ solution, 10 pmol of the generic antisense 5′UTR primer, 50 U M-MLV Reverse Transcriptase. The reverse transcription was performed at 40°C for one hour. The PCR reaction was conducted in 100 µl final volume using the entire volume of the RT reaction mixture. The PCR reaction mix was contained 10 pmol of the generic sense 5′UTR primer and 2.5 U of DuplA-Taq DNA polymerase (Zenon-Bio, Hungary). The PCR reaction was conducted under the following conditions: 1 cycle at 94°C for 1 min, 40 cycles of 94°C for 30 sec, 57°C for 30 s, 72°C for 1 min, followed by a final elongation step of 72°C for 5 min.

To determine the complete genome of a positive-sense single stranded RNA virus a series of 5′ and 3′ RACE reactions were conducted using the 3′/5′ RACE system (Roche Diagnostics, Mannheim, Germany). *The 3′ RACE* reaction in brief, cDNA was synthesized using an Oligo dT-anchor primer (Table 1) from total RNA. The Oligo dT-anchor tailed cDNA then amplified with gene-specific sense primers (s1, s2) and a PCR anchor primer (Table 1). *The 5′RACE* reaction in brief, the cDNA was generated in 20 µl final volume using oligonucleotide primers (as1a, as2a, as3a, as4a) for the four reactions from total RNA. The RNA template was degraded with RNaseH, and the cDNA was purified. The 3′end of the cDNA was polyadenilated using terminal deoxynucleotidyl transferase and dATP. The polyA tailed cDNA then amplified in 50 µl final volume using *Pfu* DNA polymerase, Oligo dT-anchor primer and primers as1b, as2b, as3b and as4b, respectively. The PCR of the 3′ and 5′ RACE experiments was conducted using the following temperature conditions: 1 cycle at 94°C for 30 sec, 35 cycles of 94°C for 35 sec, 50°C for 1 min, 72°C for 5 min, followed by a final elongation step of 72°C for 10 min. The amplicons were subjected to second PCR reaction using upstream antisense primers (as1c, as2c, as3c, as4c), the PCR anchor primer and the same PCR thermal program used in the first PCR round (Table 1). The amplification products were separated on a 1.0% agarose gel stained with ethidium bromide.

### Sequence- and phylogenetic analysis

Samples with any visible amplicons were sequenced directly with the BigDye Terminator Cycle Sequencing Ready Reaction Kit (Applied Biosystems, Warrington, UK) using the PCR primers by primer walking methods and run on an automated sequencer (ABI PRISM 310 Genetic Analyzer; Applied Biosystems, Stafford, USA). Amino acid sequences - reference strains were collected from GenBank database - were aligned by ClustalX (version 1.81) and similarity analysis were performed using GeneDoc 2.7 software [19]. Phylogenetic analysis based on amino acid alignments was conducted using the minimum evolution method of MEGA software (version 4) with poisson model [20]. The secondary structures of untranslated regions (UTR; 5′UTR, IGR and 3′UTR) were predicted using the Mfold program [21]. Percental amino acid identity of the Halastavi árva RNA virus protein regions were compared to the GenBank sequences using blastp with the following algorithm parameters: expect threshold: 10; matrix: BLOSSUM62; gap costs: existence: 11, extension: 1. The borders of the polypeptide sections were chosen by the predicted cleavage sites of the recognizable viral proteins presented in Figure 1. Complete genome and amino acid sequences of novel RNA virus (Halastavi árva RNA virus; HalV) were submitted to GenBank under accession number: JN000306.

### Nucleotide composition analysis (NCA)

A set of 352 virus complete genome or segment sequences longer than 3000 bases selected to be representative of different species, genera and families of positive-stranded RNA viruses classified in the picorna-like viruses were used for NCA [17] (sequences listed in reference [17]). Each was annotated by order, family and genus, along with host range. Mononucleotide and dinucleotide frequencies for each sequence were determined using the program “Composition Scan” in SSE version 1.0 (Simmonds, manuscript in preparation). Dinucleotide biases were determined as the ratio between the observed frequencies of each of the 16 dinucleotides from the expected frequencies determined by multiplying the frequencies of each of the two constituent mononucleotides.

NCA used the discriminant analysis program in the statistical package, SYSTAT with default parameters. Sequences were assigned to four host categories, mammal (n = 117), insect (n = 63), plant (n = 167) and fish (n = 5) and frequencies of each mononucleotide and dinucleotide used as predictive factors to infer host ranges of unknown virus sequences from the current study.

## Supporting Information

Table S1 **Host assignment of control and Halastavi árva RNA virus (HalV; JN000306) sequence by NCA.**
(DOC)
