# Supplementary material for: Novel Positive-Sense, Single-Stranded RNA (+ssRNA) Virus with Di-Cistronic Genome from Intestinal Content of Freshwater Carp (Cyprinus carpio)
Source: PLoS One. 2011 Dec 16;6(12):e29145. doi: 10.1371/journal.pone.0029145 (PMC3241695; doi:10.1371/journal.pone.0029145)
Supplement: Table S1 — Host assignment of control and Halastavi árva RNA virus (HalV; JN000306) sequence by NCA. (DOC) [file pone.0029145.s001.doc]

Table S1.

**Insect Plant Mammal Fish Correct**

Insect 59 4 0 3 89%

Plant 3 162 6 0 94%

Mammal 1 1 111 0 98%

Fish 0 0 0 5 100%

*Total 63 167 117 5 95%*

HalV 0 0 0 1
